# Scaling up self-stratifying supercapacitive microbial fuel cell

**DOI:** 10.1016/j.ijhydene.2020.06.070

**Published:** 2020-09-21

**Authors:** Xavier Alexis Walter, Carlo Santoro, John Greenman, Ioannis Ieropoulos

**Affiliations:** a Bristol BioEnergy Centre, Bristol Robotics Laboratory, UWE, T-Block Coldharbour Lane, Bristol, BS16 1QY, UK; b Biological, Biomedical and Analytical Sciences, UWE, Coldharbour Lane, Bristol, BS16 1QY, UK

**Keywords:** Microbial fuel cell, Supercapacitor, Self-powered, High power density, Urine

## Abstract

Self-stratifying microbial fuel cells with three different electrodes sizes and volumes were operated in supercapacitive mode. As the electrodes size increased, the equivalent series resistance decreased, and the overall power was enhanced (small: ESR = 7.2 Ω and P_*max*_ = 13 mW; large: ESR = 4.2 Ω and P_*max*_ = 22 mW). Power density referred to cathode geometric surface area and displacement volume of the electrolyte in the reactors. With regards to the electrode wet surface area, the large size electrodes (L-MFC) displayed the lowest power density (460 μW cm^−2^) whilst the small and medium size electrodes (S-MFC, M-MFC) showed higher densities (668 μW cm^−2^ and 633 μW cm^−2^, respectively). With regard to the volumetric power densities the S-MFC, the M-MFC and the L-MFC had similar values (264 μW mL^−1^, 265 μW mL^−1^ and 249 μW cm^−1^, respectively). Power density normalised in terms of carbon weight utilised for fabricating MFC cathodes-electrodes showed high output for smaller electrode size MFC (5811 μW g^−1^-C- and 3270 μW g^−1^-C- for the S-MFC and L-MFC, respectively) due to the fact that electrodes were optimised for MFC operations and not supercapacitive discharges. Apparent capacitance was high at lower current pulses suggesting high faradaic contribution. The electrostatic contribution detected at high current pulses was quite low. The results obtained give rise to important possibilities of performance improvements by optimising the device design and the electrode fabrication.

## Highlights

• Self-stratifying microbial fuel cells were operated in supercapacitive mode.
• Three different electrodes sizes were investigated.
• Equivalent series resistance decreased with increased size.
• Overall power increased with electrodes size while power density decreased.
• Apparent capacitance increased at low current pulses.

## Introduction

An exciting and environmentally friendly way of treating wastewater is through the utilisation of bioelectrochemical systems (BESs) [[1], [2], [3], [4]]. Among this group of technologies, microbial fuel cell (MFC) is capable of transforming organics into useful electricity [[1], [2], [3], [4], [5], [6], [7], [8]]. Particularly, MFC is composed of two electrodes (anode and cathode) in which red-ox reactions occur [[1], [2], [3], [4]]. On the anode, oxidation reaction takes place, which is facilitated by electroactive bacteria [[1], [2], [3], [4]]. These species of microorganism are capable of transferring electrons to the electrode directly or indirectly [9,10]. It was shown that these bacteria are capable of oxidising various simple and complex organic compounds, generating electricity as a by-product [11,12]. On the cathode, a reduction reaction occurs on a catalyst and an oxidant is reduced, thereby closing the circuit and electricity is produced [[13], [14], [15], [16], [17]]. Despite several oxidants being presented over the years [18], oxygen is the most commonly used because of its abundance and therefore does not need to be replaced, has high red-ox potential and is not harmful or expensive [19,20].

The first microbial fuel cell was presented over one hundred years ago [21] and several advancements have been achieved in previous years [22,23]; in the past 10–15 years, the research related to BESs and MFCs has grown exponentially [1]. One of the main bottlenecks of the technology dedicated to produce useful electricity is the low current/power generation generated. Strategies have therefore been implemented in order to boost up MFC performance [24,25]. Series and parallel connections of multiple MFCs is one way of increasing output levels [25]. Particularly, a series connection can occur when two or more MFCs are hydraulically disconnected and this operation allows increasing the operating voltage. Parallel connection instead ensures higher current generation and it can be applied also where units are hydraulically connected. Another way for improving performance is coupling the MFCs with power management systems containing energy storage devices such as capacitors, supercapacitors and batteries [24]. The first examples of coupling MFCs with external storage devices (e.g. batteries and supercapacitors) with successful energy harvesting and utilisation come from the field of robotics with “Gastrobot” [26] and the family of “EcoBots” [[27], [28], [29]]. A recent review analyses each component of the power management systems and their combinations, highlighting advantages and disadvantages [14]. Power management systems allow improvement of both voltage and current output and consequently also the resultant power generated [14,30].

Recently, capacitive behaviours regarding anode, cathode and overall MFC system were investigated [[31], [32], [33], [34], [35], [36], [37], [38], [39], [40], [41]]. The capacitive features of the anode were deeply investigated by increasing substantially the anode surface area [[31], [32], [33], [34], [35],^42^]. MFCs were also operated in supercapacitive mode and discharged galvanostatically [[36], [37], [38], [39], [40], [41],^43^,^44^]. In fact, the anode and the cathode of the MFC, charged negatively and positively by the different established environments (anaerobic and aerobic/anoxic) were considered like electrodes of an internal supercapacitor, whereby an electrochemical double layer (EDL) is formed on the electrodes surface. The anode is charged negatively and therefore attracts positive ions from the electrolyte. In parallel, the cathode charged positively attracts negative ions from the solution. The charges of the electrodes are counterbalanced by ions with opposite charge present in the solution. MFCs can be discharged by fast electrostatic processes. The charges are released in the bulk electrolyte and the charges of the electrodes surface are neutralised. The energy stored electrostatically is then delivered through galvanostatic discharges (GLV) and high-power output is obtained. Different supercapacitive MFCs have been explored in the past years varying the: i) electrode size [45], ii) cathode catalysts [38,46], iii) size (from 0.5 mL [47] to 1 L [41,46]) and iv) design [48].

**Fig. 1:**
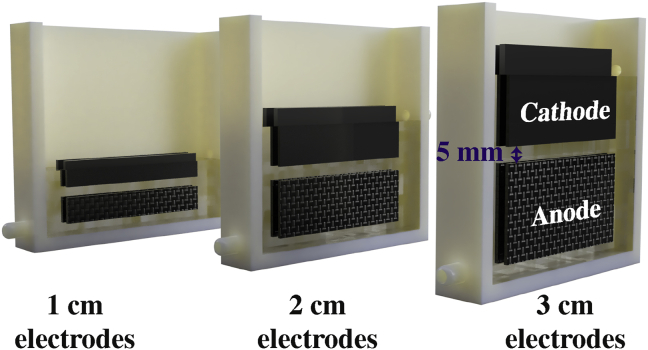
3D illustration of the SS-MFC employed in the current experiment.

This work has the goal of studying the potential of scaling up a self-stratifying MFC [[49], [50], [51]] operating in supercapacitive mode (SS-MFC). A SS-MFC exploit a phenomenon observed in any liquid column colonised by life, the naturally occurring chemical and biological stratification of the column under biological activity. SS-MFCs rely on the bio-electro-stratification of the electrolyte, here urine [52]. Hence, the steepness of the gradients and the localisation of the redoxcline are dictated by the electrodes. In SS-MFCs, cathodes are immersed in the electrolyte to about ¾ of their height [51] and are positioned around 2–8 mm above the anodes, which are occupying the bottom layer of the electrolyte (Fig. 1). The electrodes influence the natural redox gradient of the urine column, whilst applying a selecting pressure on the microbial communities, thus leading to specific bioelectrochemical equilibrium. Particularly, the organic molecules in the hydrolysed urine are used as anodic substrate for the oxidation reaction [53], while oxygen is reduced at the cathode. Here, the SS-MFC had increasing electrodes size, from 6 cm^2^ to 12 cm^2^ and 18 cm^2^ geometric surface area for an individual electrode, and reactor volume, from 49 mL to 68 mL and 88 mL. Once steady states were reached under constant load, the SS-MFC was discharged galvanostatically and parameters interest such as pulse current (i_pulse_), time of discharge (t_pulse_), equivalent series resistance (ESR), apparent capacitance, energy and power were measured. The variation of the parameters due to SS-MFC scaling up was also assessed.

## Materials and methods

### Microbial fuel cell construction

Self-stratifying MFCs were built as previously described [54,55]. Particularly, the MFCs had carbon veil wrapped over a stainless steel (SS) frame operating as anode electrodes (x2). The cathodes (x2) were fabricated by hot pressing over a stainless-steel frame (as current collector) a mixture of activated carbon (AC) and polytetrafluoroethylene (PTFE) using a pasta-maker. The anodes were inserted on the bottom of the plastic rectangular-shape container, while the cathodes were positioned on the top above the anode with ¾ of the surface area immersed and ¼ exposed to air. It was shown before that this immersed/exposed ratio was the most efficient [51]. The distance between anode and cathode was 0.5 cm. Three different MFC sizes were built and investigated as summarised in Table 1. The smaller size (S-MFC) had each anode fabricated by folding an overall area of carbon veil (10 gsm) of 180 cm^2^ around the SS frame down to a geometric area of 6 cm^2^ (1 cm × 6 cm). Each cathode had a geometric area of 6 cm^2^. The electrolyte displacement volume of S-MFC was 49 mL. The medium size (M-MFC) had an anode geometric area of 12 cm^2^ as result of an overall area of carbon veil (10 gsm) of 360 cm^2^ folded around the SS frame. All the anodes had a final thickness of about 6–7 mm. The cathodes of M-MFC had a geometric area of 12 cm^2^. The volume of M-MFC was 68 mL. The larger MFC named as L-MFC had a displacement volume of 88 mL, a geometric anode area of 18 cm^2^ (total of 540 cm^2^, 10 gsm) and a cathode geometric area of 18 cm^2^. The cathode wet surface area was measured once the SS-MFCs were running at steady state. The total cathode area takes in account the fact that a SS-MFC had two cathodes and both sides were in contact with the electrolyte.

**Table 1:** Summary of the SS-MFCs used during this experimentation.

	Cathode total surface area (cm^2^)	Cathode total wet surface area (cm^2^)	Anode total geometric surface area (cm^2^)	Anode total surface area (cm^2^)	Displacement volume (ml)	Hydraulic retention time (h)	Cathode carbon loading (g)
S-MFC	24	19.4 ± 0.4	24	180	49 ± 2	37.72	2.23 ± 0.84
M-MFC	48	28.4 ± 1.1	48	360	68 ± 2	38.73	4.47 ± 0.84
L-MFC	72	47.6 ± 1.4	72	540	88 ± 1	39.10	6.70 ± 0.84

### Operation

MFCs were inoculated using 50% in volume of partially hydrolysed urine and 50% in volume of urine effluent from existing running MFCs. After leaving the MFCs in open circuit voltage (OCV) for 10 h, the cells were connected to an external device that was keeping constant the operating voltage at 400 mV [55]. Current produced was measured constantly. After the current output was stable, the SS-MFCs were fed with partially hydrolysed and undiluted urine [53]. The urine was collected from a collection tank installed behind a urinal of the men restroom, in our laboratory. Since the collected urine already went through a partial hydrolysis (HRT of roughly 1 day in the collection tank), the collected urine had a pH of 8.8–9.2 and a solution conductivity of 27–30 mS cm^−1^ and was fed continuously through a peristaltic pump. Due to the difference in volume, peristaltic tubbings of different diameters were used to maintain similar retention times across the reactors (Table 1). S-MFC had a hydraulic retention time (HRT) of 37.72 h. At the same time, M-MFC had an HRT of 38.73 h. With respect to the L-MFC, the HRT was of 39.10 h. The MFCs were run for over 3 months in continuous mode before being investigated in supercapacitive mode.

### Electrochemical measurements

S-MFCs, M-MFCs and L-MFCs were studied in supercapacitive mode. Particularly, galvanostatic (GLV) discharges at different current pulses (i_pulse_) were performed. Initially, the MFCs were left in rest till a stable voltage was reached. This voltage corresponded to the OCV and was named as **V**_**max,OC**_. Once the GLV was initiated, the voltage dropped due to the ohmic losses of the system (anode, cathode and electrolyte). This vertical drop is **ΔV**_**ohmic**_ and after this vertical drop, the voltage reached a new value named **V**_**max**_, which is the maximum operating value of the supercapacitive MFC. Equivalent series resistance (ESR) of the cell can be calculated according to eq. (1) and represent the ohmic losses of the system:

(1) $$ESR=\;\frac{\Delta V_{ohmic}}{i_{pulse}}$$

If a reference electrode is used and it is inserted at equal distance between anode and cathode electrode in order to equally distribute the ohmic resistance of the electrolyte, the ohmic resistance of the anode and cathode can be also measured according to eqs (2), (3), respectively:

(2) $$R_{A}=\;\frac{\Delta V_{ohmicanode}}{i_{pulse}}$$

(3) $$R_{C}=\;\frac{\Delta V_{\mathrm{ohmic,cathode}}}{i_{pulse}}$$

Once the voltage reaches **V**_**max**_, the cell voltage/electrodes potential continue to decrease roughly linearly with time. The slope of the discharge named as **s** is identified as the variation of voltage over time $\left(\frac{\Delta V}{\Delta t}\right)$. The capacitance (**C**_**cell**_) is defined according to eq. (4):

(4) $$C_{\mathrm{cell}}=\;\frac{i_{\mathrm{pulse}}}{s}=\;\frac{i_{\mathrm{pulse}}}{\frac{\Delta V}{\Delta t}}$$

The capacitance of anode and cathode named as **C**_**A**_ and **C**_**C**_ can also be calculated according to eq. (4) but considering the variation of the electrode potential over time. The capacitance is a parameter of interest in electrical double layer (DL) capacitor where the electrostatic contribution is present. It was shown that in supercapacitive MFCs, especially at low current pulse, two contributions were considered, the first electrostatic due to the double layer and the second one due to the red ox reactions occurring on the MFC electrodes [39]. Therefore, especially at low current pulses, it is more correct to utilise the term “apparent” capacitance since it is practically impossible to discriminate the two contributions. The maximum power achievable was calculated according to eq. (5).

(5) $$P_{\max}=\;V_{\max}\;\times \;i_{\mathrm{pulse}}$$

Pulse power (**P**_**pulse**_) is defined according to eq. (6) and the ratio between the pulse energy (**E**_**pulse**_) and the time of the pulse:

(6) $$P_{\mathrm{pulse}}=\;\frac{E_{\mathrm{pulse}}}{t_{\mathrm{pulse}}}$$

The energy delivered during the pulse is defined as the product of i_pulse_ and the integral under the discharge curve (V-time) during the t_pulse_ according to eq. (7):

(7) $$E_{\mathrm{pulse}}=i\;\times \;\int_{0}^{t}V\;dt$$

## Results and discussion

### Galvanostatic discharges at t_pulse_ 5 s

**Fig. 2:**
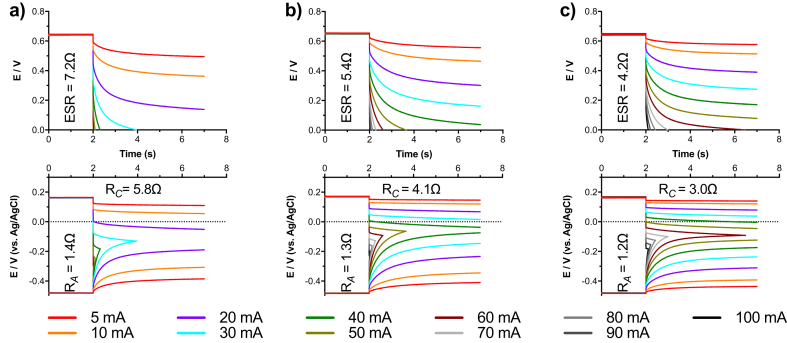
Cell Voltage (above) and Electrode Potentials (below) during 5 s discharges at different i_pulse_ for S-MFC (a), M-MFC (b) and L-MFC (c).

Galvanostatic discharges at t_pulse_ of 5 s were performed and cell voltage profile and anode/cathode potential profiles were presented in Fig. 2. Same galvanostatic discharges plots were also summarised in the Supporting Information reporting the current density, normalised by the cathodes wet surface area, used during discharges (Figs. S1, S2, S3). ESR, R_A_ and R_C_ were calculated from GLV discharges. ESR decreased with increasing size of the SS-MFC and measured to be 4.2 Ω, 5.4 Ω and 7.2 Ω for L-MFC, M-MFC and S-MFC respectively. Concerning L-MFC, R_A_ was 1.2 Ω and R_C_ was 3 Ω, corresponding to 29% and 71% of the overall ESR. R_A_ and R_C_ were slightly higher for M-MFC measuring 1.3 Ω and 4.1 Ω respectively and were 24% and 76% of the total ESR. The S-MFC had the higher ESR and R_A_ and R_C_ contributions were 19% and 81% of the total measuring 1.4 Ω and 5.8 Ω respectively. These results were expected since the increasing of the size of the electrodes, results in an overall decrease in the ohmic resistance of the electrodes. Lower ESR led to longer times for total discharge. In fact, it can be noticed that the larger is the SC-MFC, the longer are the discharges (Fig. 2). For example, considering i_pulse_ of 60 mA, the t_pulse_ for total discharge was 4.48 s, 0.62 s and 0.02 s for L-MFC, M-MFC and S-MFC respectively. The time of complete discharge associated to i_pulse_ and the three different configurations is summarised in Table 2. Qualitatively, it can also be noticed that the apparent capacitance of the SC-MFC increased with the size of the SC-MFC; in fact, it can be seen that the slope decreases with larger electrodes. Also, this result is expected because larger electrodes create a greater electrostatic double layer on the interface.

**Table 2:** Time of complete discharge associated to different i_pulses_.

i_pulse_ (mA)	S-MFC (s)	M-MFC (s)	L-MFC (s)
20	43.276	124.999	–
30	1.959	61.026	–
35	–	–	206.633
40	0.318	4.999	–
50	0.093	1.66	27.181
60	0.019	0.619	4.48
70		0.286	0.994
80		0.137	0.427
90		0.06	0.213
100		0.022	0.106
110		0.003	0.049

### Maximum power and pulsed power

**Fig. 3:**
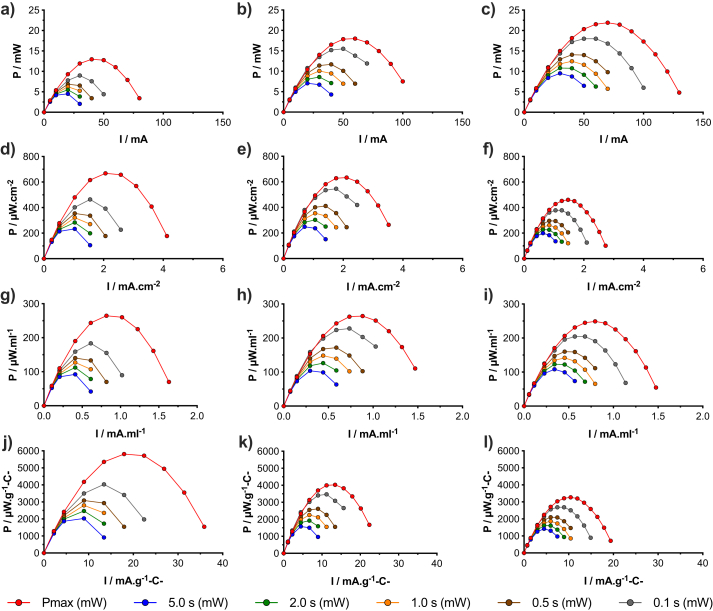
*P*_max_*and P*_*pulses*_ of supercapacitive SS-MFC (a), M-MFC (b) and L-MFC (c). Power density considering the area of the cathode exposed to the electrolyte for S-MFC (d), M-MFC (e) and L-MFC (f). Volumetric power density for S-MFC (g), M-MFC (h) and L-MFC (i). Power density referred to the carbon loading of the cathode electrodes for S-MFC (j), M-MFC (k) and L-MFC (l).

GLV discharges at t_pulse_ of 5 s were necessary for determining ESR and therefore calculating P_max_ as well as for calculating the P_pulse_ at t_pulse_ of 5 s, 2 s, 1 s, 0.5 s and 0.1 s. P_max_ and P_pulse_ at different t_pulse_ were shown in Fig. 3. Power curves are presented in terms of absolute power produced (mW) (Fig. 3 a-c), and also normalised as power density with respect to: (i) the cathode exposed to the electrolyte (μW cm^−2^) (Fig. 3 d-f); (ii) the volume of the electrolyte within the reactor (μW mL^−1^) (Fig. 3 g-i) and (iii) the carbon loading of the cathodes electrodes (μW gC^−1^) (Fig. 3 j-l).

Overall P_max_ increased with SC-MFC size, measuring as peak 21.91 mW, 18.00 mW and 12.96 mW for L-MFC, M-MFC and S-MFC respectively. These results were again expected as more electrode area is actively involved in the red-ox reactions occurring on the two electrodes. Concerning the power density referred to cathode geometric area, the smaller the MFC the better performance was achieved in terms of P_max_. Particularly, P_max_ was 460 μW cm^−2^, 634 μW cm^−2^ and 668 μW cm^−2^ for L-MFC, M-MFC and S-MFC respectively. P_max_ does not consider the capacitance of the electrodes i.e. the lowering in voltage/potentials after reaching V_max_.

Focusing on P_pulse_, the best performing SC-MFC was M-MFC, with peak of P_pulse_ of 545 μW cm^−2^ (0.1 s), 411 μW cm^−2^ (0.5 s), 354 μW cm^−2^ (1 s), 302 μW cm^−2^ (2 s) and 248 μW cm^−2^ (5 s). Similarly, considering the peak of P_pulse_ expressed against the volume of the electrolyte (μW mL^−1^), M-MFC had the highest value of a volumetric P_pulse_ of 228 μW mL^−1^ (0.1 s), 172 μW mL^−1^ (0.5 s), 148 μW mL^−1^ (1 s), 126 μW mL^−1^ (2 s) and 103 μW mL^−1^ (5 s).

These results are quite interesting since generally, the smaller is the MFC the higher is the power density both referred to cathode geometric area and volume of the reactor [56]. MFCs are operating on the basis of two distinct environments being established. A strict anoxic or anaerobic environment is needed for electroactive bacteria to biocatalyse the oxidation reaction at the anode. The presence of oxygen may be detrimental for the growth of the biofilm and the establishment of an efficient electroactive anodic biofilm. An oxic or aerobic environment is instead needed at the cathode for the reduction reaction. This equilibrium is very important in order to avoid mixed potentials and perturbation of established environments that might lower the voltage/potential output. The smaller the MFC system becomes, the more difficult it is to maintain separation between the two environments. In self-stratifying MFCs, the separation of environments relies on the stratification occurring naturally in a urine column in which bacteria separate the bottom part (anaerobic/anoxic) from the top part (aerobic/oxic). If the height of the column is too small, no clear separation is achieved, and the anaerobic environment might be compromised. This aspect has been discussed recently [55] where the smaller underperformed compared to M-MFC and L-MFC. The anode performance seemed to be the most affected with anodic polarization curves clearly indicating limitations of this electrode performance [55]. The anode performance is mainly responsible for contributing to the apparent capacitance especially on the faradaic contribution. The lower the anode performance, the lower the apparent capacitance and consequently the discharges profile and in turn also the maximum power and the P_pulse_ as shown previously [47].

Another important method of expressing the power generated is through the normalisation for the carbon loading of the cathode electrodes. In this case, S-MFC has the highest P_max_ and P_pulse_ measuring 5811 μW gC^−1^ (P_max_), 4036 μW gC^−1^ (0.1 s), 3085 μW gC^−1^ (0.5 s), 2798 μW gC^−1^ (1 s), 2466 μW gC^−1^ (2 s) and 2031 μW gC^−1^ (5 s). The explanation for these results can be attributed to the difference in electrode structures between MFCs and supercapacitors. In fact, for MFCs, the electrodes are fabricated in order to facilitate the reactions occurring at both electrodes. Considering the anode, a three-dimensional (3D) structure is preferred in order to accommodate the greatest electroactive bacteria as possible that act as biocatalyst. The 3D structure is also useful for allowing reagents diffusion/perfusion and reaction products expulsion. The cathode structure is also fabricated in order to accelerate the oxygen reduction reaction (ORR) using a 3D pellet-type air-breathing cathode with the intention of creating hydrophilic/hydrophobic interfaces. This interface allows for a desired three phase interface (TPI) in which oxygen in gas phase, proton within the liquid electrolyte and electrons moving into the solid electrode contribute to the ORR. Optimised SC electrodes can be considered as 2D electrodes in which high surface area materials are deposited through coating, drop casting, etc on a conductive current collector. This allows the creation of an electrical double layer capacitor (EDLC) and the electrode surface area per volume of device is optimised. As the EDL occurs on the surface of the electrode exposed directly to the electrolyte, a complex 3D electrode as used in the anode and the cathode of an MFC is not needed. After these considerations, it can be concluded that the electrode weight of MFCs and therefore the carbon material utilised for the formation of the electrodes is actually very much overestimated compared to the real need of creation of an EDL. Therefore, larger electrodes, would inevitably carry more carbon loading but of which not all will be actively involved in the formation of an EDL and consequently not utilised successfully during galvanostatic discharges.

### Apparent capacitance

During discharges of the SC-MFCs, two different phenomena occur simultaneously on the interface between electrode and electrolyte. Firstly, the electrostatic double layer formed on the electrode surface is discharged and the counterions attracted by the charged electrodes are released into the electrolyte. Secondly, in parallel, a red-ox reaction occurs on the electrode surface. Both electrostatic double layer (electrostatic contribution) and red-ox reactions (faradaic contribution) are involved in the capacitance measured. Electrostatic and faradaic processes retain different kinetics. Generally, the kinetic related to electrostatic processes is much faster compared to the one delivered by faradaic processes. Therefore, it can be speculated that: i) high current pulses (short t_pulse_) will be associated to electrostatic processes; ii) low current pulses (long t_pulse_) will be associated to faradaic processes [39].

In a traditional EDLC, the voltage decreases linearly with the current and the output appears as a straight line. This trend can be noticed only in high current/very short t_pulse_ (Fig. 2). As the i_pulses_ goes towards lower values, the voltage/potential profile becomes non-linear after an initial linear decrease (Fig. 2). These trends well fit with the explanation described above. It is normally extremely difficult to identify and quantify the two different contributions [39,43].

**Fig. 4:**
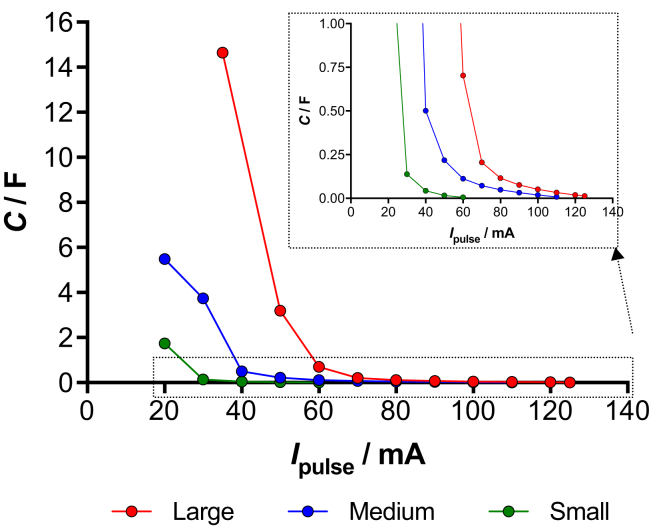
Apparent capacitance of supercapacitive S-MFC, M-MFC and L-MFC.

Apparent capacitance against i_pulse_ of S-MFC, M-MFC and L-MFC are plotted in Fig. 4. It can be seen that for high current pulses, the apparent capacitance is quite low at ≈0.1 F. This can be attributed to the pure electrostatic contribution. At lower i_pulse_, the apparent capacitance increased significantly with the higher values recorded at 14.65 F (i_pulse_ = 35 mA, L-MFC), 5.49 F (i_pulse_ = 20 mA, M-MFC) and 1.74 F (i_pulse_ = 20 mA, M-MFC). These values were certainly inflated importantly by the faradaic contribution, which at low current pulses becomes the predominant contributor to the apparent capacitance.

## Overview and outlook

Three different size SC-MFCs were operated in supercapacitive mode and discharged galvanostatically. ESR decreases with the increasing in size. Simultaneously also R_A_ and R_C_ decreased with the increasing in electrode sizes. Overall power increases with the size but not proportionally once related to the electrode size and the SC-MFC volume. The medium size electrodes MFC was found to be the best performing in terms of power density (cathode surface area and MFC electrolyte volume). Similar results were found in the same MFCs operating under constant external voltage. In fact, the polarization tests conducted on the anode electrodes showed that the smaller electrode suffered from an incomplete anaerobiosis lowering the performance and perform less than the medium and large size electrodes [55]. It was speculated that this might be due to the lower column height above the small anode electrode. Higher power output was achieved by S-MFC once normalised by the carbon utilised for fabricating the electrodes. This was the result of electrodes not being optimised for supercapacitive operations but adopted for MFC electrodes. The results related to the apparent capacitance are also important for better understanding the electrostatic and faradaic contributions despite the difficulties in discriminating the two contributions.

The results obtained in this work are of extreme importance for learning the limitations of the system and provide useful suggestions for further improvements. Self-stratifying MFC system is able to naturally separate oxic/aerobic and anoxic/anaerobic environments. Therefore, a correct separation between anode and cathode has to be adopted as well as a column of electrolyte above the anode need to be selected in order to confine and maintain the anaerobic conditions for a correct functioning of the anode electrode. A greater area to volume ratio should also be adopted in order to increase the active area to deliver useful energy/power, therefore more electrodes should be packed in the same volume but guaranteeing electrolyte/reagents diffusion/perfusion. In order to enhance the current/power, the electrode fabrication should be optimised for increasing the electrostatic double layer formation and not just fulfil MFC requirements. Therefore, thinner layers should be used for fabricating electrodes, which should become thinner and thinner and moving towards the electrodes of supercapacitors. A transition from 3D electrodes to a 2D electrode might be envisioned and encouraged to pursue for boosting up the electrochemical performance.

## Conclusions

Self-stratifying MFCs with different size in terms of electrode geometric area and system volume were investigated in supercapacitive mode. SC-MFC were fuelled with hydrolysed human urine. The larger the electrode size the lower was the overall ohmic resistance of the system. Higher power output was recorded with larger electrode sizes: P_max_ = 21.9 mW (L-MFC), P_max_ = 18.0 mW (M-MFC) and P_max_ = 13.0 mW (S-MFC). Considering power density, medium electrode size SC-MFC had the higher value probably due to the anode limitation at smaller scale where the total anaerobic environment might have been compromised and affected negatively the biofilm development. The normalisation in function of the quantity of electrodes utilised (in weight), showed that the smaller the SC-MFC the higher was the power output. This was due to the utilisation of 3D electrode structures, which are not optimised for supercapacitive features. Analysis on the apparent capacitance underlines two different behaviours with electrostatic contribution being predominant at high i_pulse_ (short t_pulse_) and faradaic contribution being predominant at low i_pulse_ (long t_pulse_).

## Declaration of competing interest

The authors declare that they have no known competing financial interests or personal relationships that could have appeared to influence the work reported in this paper.
