# Supplementary material for: Scaling up self-stratifying supercapacitive microbial fuel cell
Source: Int J Hydrogen Energy. 2020 Sep 21;45(46):25240–8. doi: 10.1016/j.ijhydene.2020.06.070 (PMC7491701; doi:10.1016/j.ijhydene.2020.06.070)
Supplement: Multimedia component 1 [file mmc1.docx]

**Supporting Information**

**Scaling up self-stratifying supercapacitive microbial fuel cell**

*Xavier Alexis Walter^1^, Carlo Santoro^1,+^, John Greenman^1,2^, **Ioannis Ieropoulos^1^

^1^ Bristol BioEnergy Centre, Bristol Robotics Laboratory, T-Block, UWE, Coldharbour Lane, Bristol, BS16 1QY, UK

^2^ Biological, Biomedical and Analytical Sciences, UWE, Coldharbour Lane, Bristol, BS16 1QY, UK

^+^ Currently: School of Chemical Engineering and Analytical Science, The University of Manchester, Sackville Street, The Mill, M13AL, United Kingdom

Corresponding authors

* Xavier Alexis Walter. E-mail: xavier.walter@uwe.ac.uk

** Ioannis Ieropoulos. E-mail: [ioannis.Ieropoulos@brl.ac.uk](mailto:ioannis.Ieropoulos@brl.ac.uk)

**Figure S1.** *Cell Voltage (above) and Electrode Potentials (below) during 5 s discharges at different i_pulse_ density for S-MFC*

**Figure S2.** *Cell Voltage (above) and Electrode Potentials (below) during 5 s discharges at different i_pulse_ density for M-MFC*

**

**Figure S3.** *Cell Voltage (above) and Electrode Potentials (below) during 5 s discharges at different i_pulse_ density for L-MFC*

**

**Figure S4.** *Cell Voltage (above) and Electrode Potentials (below) during completely discharges at different i_pulse_ for S-MFC (a), M-MFC (b) and L-MFC (c). *For the L-MFC the discharge was at i_pulse_ = 35 mA.*
